# Characterizing the Brain Structural Adaptations Across the Motherhood Transition

**DOI:** 10.3389/fgwh.2021.742775

**Published:** 2021-10-07

**Authors:** Magdalena Martínez-García, María Paternina-Die, Manuel Desco, Oscar Vilarroya, Susanna Carmona

**Affiliations:** ^1^Instituto de Investigación Sanitaria Gregorio Marañón, Madrid, Spain; ^2^Centro de Investigación Biomédica en Red de Salud Mental (CIBERSAM), Madrid, Spain; ^3^Departamento de Bioingeniería e Ingeniería Aeroespacial, Universidad Carlos III de Madrid, Madrid, Spain; ^4^Centro Nacional de Investigaciones Cardiovasculares (CNIC), Madrid, Spain; ^5^Department of Psychiatry and Legal Medicine, Autonomous University of Barcelona, Barcelona, Spain; ^6^Hospital del Mar Medical Research Institute, Barcelona, Spain

**Keywords:** mother, neuroplasticity, MRI, maternal behavior, pregnancy

## Abstract

Women that become mothers face notable physiological adaptations during this life-period. Neuroimaging studies of the last decade have provided grounded evidence that women's brains structurally change across the transition into motherhood. The characterization of this brain remodeling is currently in its early years of research. The current article reviews this scientific field by focusing on our longitudinal (pre-to-post pregnancy) Magnetic Resonance Imaging (MRI) studies in first-time parents and other longitudinal and cross-sectional studies of parents. We present the questions that are currently being answered by the parental brain literature and point out those that have not yet been explored. We also highlight potential confounding variables that need to be considered when analyzing and interpreting brain changes observed during motherhood.

## Introduction

Motherhood is a transformative experience for women. It confers substantial anatomical and physiological changes in the endocrine, cardiovascular, respiratory, renal, and musculoskeletal systems of the mother (1). Neuroimaging studies of the last decade have confirmed that the women's brain also undergoes visible structural changes during the motherhood transition. However, the nature of this brain remodeling is still in its early years of research.

In 2017, our group led one of the most notable longitudinal projects on this behalf: a study that tracked the neuroanatomical MRI data of primiparous women across three sessions: a few months before their first pregnancy, during the early postpartum, and at 2 years after parturition, using fathers and non-parents as comparison groups (2). This study found prominent pre-to-post pregnancy volumetric Gray Matter (GM) reductions in theory-of-mind regions in first-time mothers. Since then, we have conducted several pre-to-post pregnancy longitudinal MRI studies to characterize the initially observed GM reductions and understand the potential underlying mechanisms. Here, we cover the brain structural findings that we have found so far and discuss variables that need to be considered to correctly interpret brain changes during the motherhood transition. The manuscript is divided into the main questions that our research group and other groups have aimed to directly or indirectly respond to through structural MRI. First, we summarize the neuroanatomic changes that have been detected through the motherhood transition. Then, we discuss the following topics: 1) which are the mediating factors behind these changes? 2) which are the underlying neural mechanisms? 3) what do we know about the changes' temporal course? and, 4) are deviations of these adaptations related to the emergence of postpartum mental health disorders? We also contemplate other factors not directly related to motherhood as potential confounders of the observed changes. This review article aims to provide cohesion to the structural parental brain literature and set future directions for those critical questions that remain unresolved.

For the sake of efficiency, during the manuscript, we will refer to mothers as persons who identify as women that undergo pregnancy through natural or assisted processes. Future studies need to address the unique brain processes of mothers that do not undergo pregnancy.

## Neuroanatomic Changes During the Transition to Motherhood

**Figure 1:**
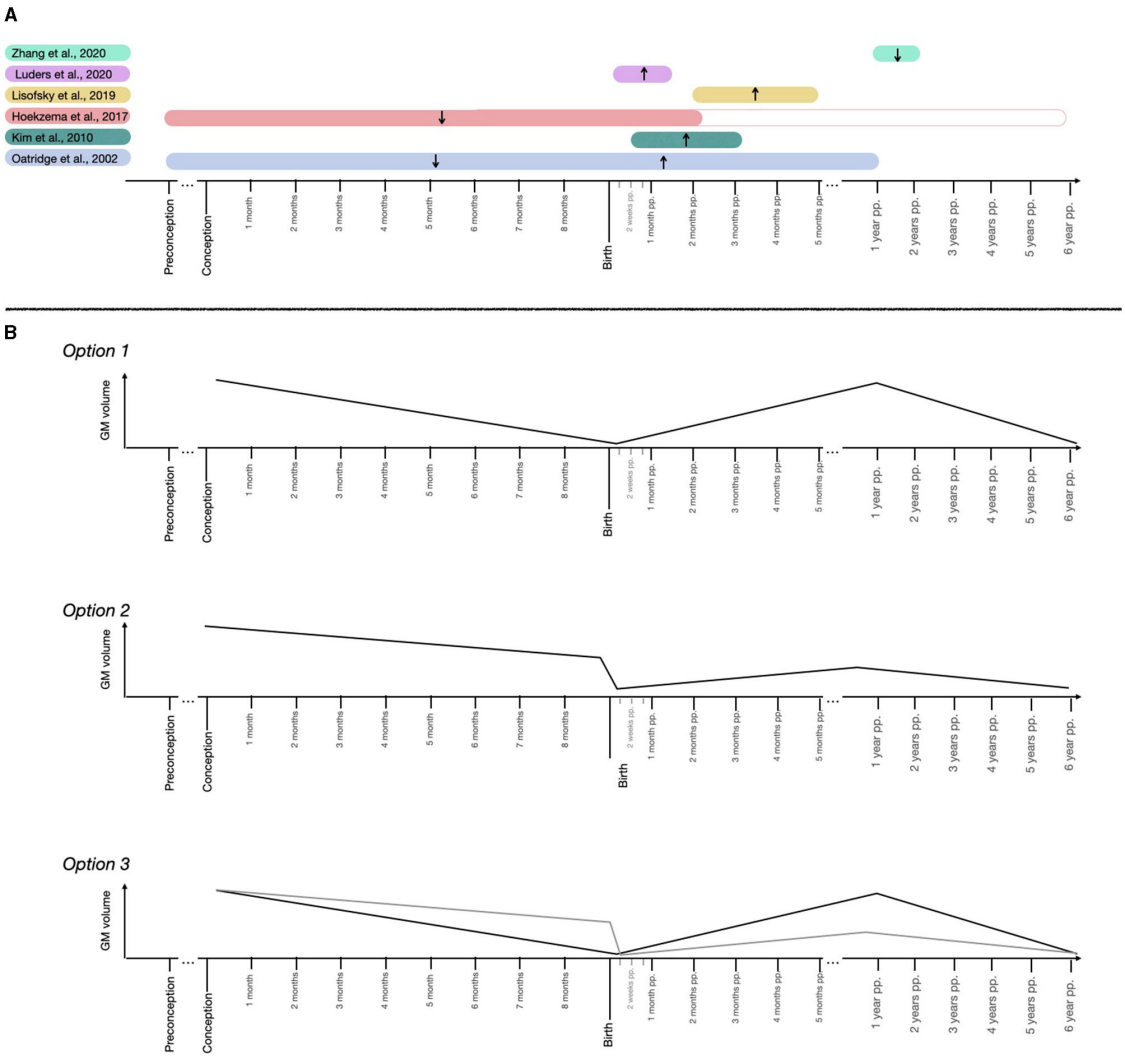
Schematic representation of the maternal brain GM volume findings and suggested trajectories. **(A)** Findings of the longitudinal MRI structural studies tracking the maternal brain. Represented studies are (2–7). **(B)** Hypothetical trajectories in GM volume across pregnancy and postpartum periods.

Existing human neuroimaging studies have applied both longitudinal and cross-sectional designs to track the short-term effects of pregnancy on the brain. Figure 1A and Table 1 sum up and outline the most relevant findings from structural studies that track brain changes during the transition to motherhood.

**Table 1:** Summary of the characteristics and findings of the longitudinal MRI structural studies tracking the maternal brain.

	**Oatridge et al. (7)**	**Kim et al. (6)**	**Hoekzema et al. (2)**	**Lisofsky et al. (5)**	**Luders et at. (4)**	**Zhang et al. (3)**
Period ^*^	Pre-pregnancy (*n* = 2) ➤22.5 weeks gestation (*n* = 4) ➤before delivery (*n* = 9) ➤6 weeks pp (*n* = 9) ➤24 weeks pp (*n* = 7) ➤40 weeks pp (*n* = 3) ➤1 year pp (*n* = 3)	3 weeks pp ➤3.5 months pp	Pre-pregnancy ➤2 months pp	2 months pp ➤5 months pp	1.5 days pp ➤5 weeks pp	1 year pp ➤2 years pp
Participants	9 mothers	19 mothers	25 mothers and 25 nulliparous controls	24 mothers and 24 nulliparous women	14 mothers	21 mothers
Primiparous	Not reported	58%	100%	100%	50%	100%
Age at baseline (mean ± sd)	Mean: 31 years. Range: 20–38 years.	33.27 ± 6.07 years	Mothers: 33.36 ± 3.97 years. Nulliparous: 31.10 ± 5.63 years	Mothers: 28.38 ± 3.41 years. Nulliparous: 25.42 ± 2.95 years	32.8 ± 4.0 years	30.03 ± 2.75 years
Processing method	Contour and thresholding semiautomatic technique	Voxel-based morphometry toolbox (SPM2)	Voxel-based morphometry toolbox (SPM12)	Voxel-based morphometry toolbox (SPM8)	Voxel-based morphometry toolbox (SPM8)	Volume in Computational Anatomy toolbox (SPM12)
Contrast and threshold	**-**	Post > Pre (*p* < 0.05 FDR-corr, extent > 100 voxels)	Mothers (Post-Pre) < Nulliparous women (Post-Pre) (peak level *p* < 0.05, FWE-corr)	Group by time interaction (*p* < 0.05 FWE-corr, exten t >200 voxels)	Post > Pre (peak level p <0.001, FWE-corr)	Post > Pre (peak level p <0.05, FDR-corr)
Direction	Brain size decreases until delivery, then increases	GM volume increases	GM volume decreases	GM volume increases	GM volume increases	GM volume decreases
**MODIFIED REGIONS**						
Frontal regions		R superior frontal	Superior medial frontal			Superior frontal
		Medial frontal				Medial frontal
		Middle frontal	L Middle frontal	Middle frontal		Middle frontal
			Medial orbitofrontal			
			L inferior orbitofrontal			
		L inferior frontal	Inferior frontal		L inferior frontal	Inferior frontal
					L Central and frontal operculum	
			L Superior Frontal	Ventro medial prefrontal		
		Precentral			R medial precentral	Precentral
						R Paracentral
Temporal regions		L superior temporal	Superior temporal			
			Middle temporal			
			Inferior temporal			
			Fusiform			
Parietal regions		Precuneus	Precuneus		R precuneus	R precuneus
		Superior parietal				L Superior parietal
		Inferior parietal				
					R middle occipital	
		Postcentral			R postcentral	Postcentral
Insular cortex		Insula	L Insula			L insula
Cingulate cortex		R anterior cingulate	Anterior/posterior cingulates	Anterior cingulate		
		Cingulate				
Subcortical regions						
			L hippocampus			
		R Parahippocampal	Parahippocampal			R Parahippocampal
				L Nucleus accumbens		
		R Thalamus			Thalamus	Thalamus
		Cerebellum		Cerebellum		R Cerebellum
		Caudate			L caudate	Caudate
		Substantia nigra				
		R amygdala				
		R Putamen				
		Mammilary body				
		R globus pallidum				
		Hypothalamus				
		L brainstem (pons, medulla)				
Additional structural findings ^**^	Ventricular size increases until delivery, then decreases		Cortical thickness and area decreases (Freesurfer v5.3) GM decreases maintained up to 2 years pp		Decreased BRAIN age index (8) R hippocampus (subiculum,CA2 and CA3) (9) and amygdala increases (10)	WM increases and cortical thickness decreases. Cross-sectional analysis mothers vs nulliparous women: lower GM and cortical thickness, higher WM and gyrification index
			GM decreases maintained up to 6 years pp (11)			
			Flattening of the cortex (12)			
			L nucleus accumbens decreases (13)			

* *Periods were reported as the mean time-points when the scans were acquired*.

** *The additional structural findings come from other studies or analyses that have used the same participants sample. pp, postpartum; FDR, false discovery rate; FWE, family wise error; GM, gray matter; WM, white matter; L, left hemisphere; R, right hemisphere; p, p-value*.

The study of the women's brain changes associated with pregnancy dates to the first half of the twentieth century. In 1931, enlargements of the pituitary gland, one of the primary sources of prolactin during pregnancy, were first observed in deceased pregnant women (14) and were later corroborated by *in-vivo* MRI of this structure (15–17). The gradual increase of the pituitary gland during pregnancy is thought to reflect an estrogen-stimulated proliferation and hypertrophy of prolactin cells (18). Besides the pituitary gland volume assessments, the first longitudinal study that tracked the whole brain found increased ventricles and reduced outer border of the brain peaking at parturition in a small sample of pregnant women (7).

In 2017, our research group longitudinally compared the brains of first-time mothers from preconception to 2 months postpartum (pre-to-post hereafter) and found pronounced GM volume reductions within cortical regions that highly overlapped with the theory-of-mind or Default Mode (DM) network (2). The modified structures included frontal and temporal areas, precuneus, left insula, anterior cingulate cortex, and left hippocampus and parahippocampal regions. Besides, the pre-to-post pregnancy cortical changes predicted the quality of mother-to-infant attachment and the absence of hostility toward their babies. Such reductions were maintained at 2 years after giving birth, except for a partial left hippocampal volume recovery (2). We then extended this research by reporting that the pre-to-post reductions reflected an overall flattening of the cortex characterized by decreases in cortical thickness, surface area, local gyrification index, sulcal depth, and sulcal length, as well as increases in sulcal width (12). In addition, a recent study using the same prospective dataset of first-time mothers reported volume reductions within the left ventral striatum –a subcortical region containing the nucleus accumbens that acts as the core of the maternal reward system (13).

Other longitudinal studies have approached this question by analyzing how the brain changes during the postpartum period (3–6, 8–10). Luders et al. (8) applied a brain age algorithm to a prospective dataset of mothers and found that mothers' brains were considerably younger at 4–6 weeks postpartum compared to at 1–2 days after childbirth and suggested that such “rejuvenation” reflected GM increases, rather than decreases. The same sample of mothers also presented GM increases within the inferior frontal, precuneus, precentral, and postcentral gyri occipital and operculum regions, and subcortical regions such as the thalamus and caudate (4), the right CA1 and CA3 hippocampal regions (9), and the superficial and centromedian regions of the amygdala (10). Kim et al. (6) scanned mothers at a later postpartum period (at 2–4 weeks and at 3–4 months after birth) and found increasing GM trends in similar frontal, parietal and subcortical areas, but the GM increases further extended to the superior temporal gyrus, cingulate cortex, insula, hypothalamus, putamen, globus pallidum, cerebellum and brainstem. In addition, the higher the volume within the subcortical regions, the higher the maternal positive perception of her baby (6). Lisofsky et al. (5) applied a similar postpartum design to Kim et al. (6) and extended it by including a control group of nulliparous women and by excluding multiparous participants. Compared to non-mothers, mothers displayed GM volume increases in anterior cingulate cortex, middle frontal and medial prefrontal regions, cerebellum, and left nucleus accumbens, from 1–2 to 4–5 months postpartum (5). Changing regions were similar to those reported by Kim et al. (6), but less in number, likely due to the inclusion of a nulliparous control group. Moreover, mothers had smaller GM volumes at 1–2 months postpartum compared to non-mothers, and this volume difference diminished at 3–4 months postpartum (5). This finding suggests that the postpartum GM increases are preceded by initial reductions, in agreement with the results of Hoekzema et al. (2). Notably, this study also found that the younger the mothers were, the stronger the GM increases from 1–2 to 3–4 months postpartum, suggesting that samples with older mothers might display different GM postpartum trajectories. Finally, Zhang et al. (3) scanned primiparous mothers at 8 months and 2 years after birth and found GM reductions and white matter (WM) increases in frontal areas, superior parietal gyrus, precuneus, and insula, WM increases in temporal regions and GM reductions in subcortical regions such as parahippocampal gyrus, thalamus, caudate, and cerebellum, which were positively associated with the mother's empathic abilities. Similarly, the authors found lower GM and higher WM volumes when comparing the mothers' initial postpartum scans with non-mothers' scans (3). Most of the changing regions coincided with the longitudinal analysis with mothers, while middle temporal, anterior cingulate, putamen, and globus pallidum were uniquely modified. This suggests that such regions might change at an earlier postpartum state [in agreement with the results of Kim et al. (6)] but are no longer modified during late postpartum.

Of note, many of the modified regions found by these longitudinal studies coincide (Table 1). Cortical regions that consistently change in mothers include frontal areas, superior temporal and parietal gyri, precuneus, postcentral, and precentral gyri, anterior cingulate cortex and insula, most of which are DM nodes, and common subcortical regions are the hippocampus, amygdala, thalamus, caudate, nucleus accumbens, and cerebellum.

Cross-sectional designs have also found GM structural variations in the maternal brain. Lisofsky et al. (19) found that, as compared to nulliparous women (*N* = 30), early postpartal first-time mothers (*N* = 30) had lower volume in the left putamen, a striatal region involved in navigation strategies. Also, the higher the prepartal estrogen levels and allocentric navigation strategy (flexible learning) vs. egocentric navigation strategy (stimulus-response learning) in mothers, the greater postpartal reductions the mothers displayed within the left putamen (19). Kim et al. (20) detected a positive association between postpartum months and prefrontal cortical thickness in a group of 39 first-time mothers with diverse socioeconomic and racial backgrounds, and these results were independent of the mother's age, race, income levels or depressive symptoms. Moreover, higher cortical thickness within those prefrontal regions was associated with higher self-reported parental efficacy (20).

Brain regions whose structure changes in mothers (Table 1) are consistent with functional modifications reported during postpartum. A group of event-related functional MR studies scanned mothers at 48 h of delivery and again at 4–7 weeks postpartum and found that the later postpartum session was characterized by lower activity during response inhibition within inferior frontal, anterior cingulate and precentral regions (21), higher emotion reactivity within the insula and frontal regions (22), and decreased reactivity during emotional anticipation in the anterior cingulate cortex (23). Also, resting-state functional MR (rsfMRI) acquisitions of first-time mothers at 1 and 2 year postpartum revealed changes in the local neural activity strength and coherence of DM regions such as precuneus, cingulate, insula, and frontal and temporal regions between these two time points (24). Finally, Dufford et al. (25) found an association between postpartum months and resting-state functional connectivity between the left amygdala and the anterior cingulate gyrus, left nucleus accumbens, caudate, right putamen, left pallidum, and left cerebellum, and between the right amygdala and the caudate. These findings indicate that structural and functional changes co-occur in overlapping DM and subcortical regions during the transition into motherhood.

Taken together, literature suggests that motherhood is associated with dynamic brain adaptations in cortical regions that overlap with the DM/ theory-of-mind network and subcortical reward regions. These adaptations seem to differ in magnitude and direction depending on the time frame studied. Specifically, longitudinal studies indicate a GM volume decrease (2, 7) and a flattening of the cortex (12) in mothers scanned before and after pregnancy, followed by volume increases during the early postpartum (4–7, 9, 10), and volume decreases again during the late postpartum (3). Finally, changes in GM volumes do not reach pre-pregnancy levels at 2 years (2) or 6 years (11) postpartum. If one were to unify these longitudinal results, several GM structural trajectories emerge as plausible, which are outlined in Figure 1B. First, GM might decrease in volume during pregnancy, increase right after birth until reaching baseline levels and fall again late postpartum (Figure 1B, Option 1). Alternatively, the prenatal GM decrease might be more abrupt during the peripartum period, and postnatal trajectories might not reach pre-pregnancy baseline levels (Figure 1B, Option 2). Another possibility would be a combination of the prenatal and postnatal trajectories reflected in Options 1 and 2 (Figure 1B, Option 3).

## Which are the Mediating Factors?

Researchers have not disentangled yet which factors trigger and coordinate the brain morphometric changes observed in mothers. Theoretically, these changes could be mediated either by intrinsic pregnancy-related hormonal factors, by extrinsic environmental factors that translate to internal signals, or a combination of both. As discussed in the previous section, the brain adaptations to motherhood are not linear but instead seem to differ during pregnancy and postpartum, two periods characterized by unique physiological events (gestation vs. lactation), endocrine fluctuations (26, 27), and immune responses (28). This difference is also observed from a psychological point of view. Whereas an expectant mother often engages in imagination and simulation, the brain of a postpartum mother often engages in executive functions for planning the tasks necessary to take care of the newborn. It is therefore reasonable to think that the factors that mediate the neural changes in mothers might be distinct before and after the birth.

### Pregnancy Hormones

The transition to motherhood is characterized by unparalleled hormonal fluctuations that orchestrate the physiological changes that mark pregnancy, parturition, and lactation (26, 27, 29, 30). Sex-steroid hormones such as progesterone and estradiol (E_2_) are mainly produced by the placenta during pregnancy (31). Accordingly, they increase steadily across the three trimesters, fall off rapidly at parturition after placental separation, and remain low during postpartum (29, 30). Oxytocin and prolactin are neurohormones synthesized by the hypothalamus and anterior pituitary and are released both centrally and peripherally from the posterior and anterior pituitary, respectively (32, 33). Oxytocin levels remain inhibited during pregnancy and rise at parturition, following a pulsatile secretion pattern to regulate uterine contractions and stimulate milk ejection (30). Prolactin levels increase during pregnancy, to prepare the breasts for lactation (29, 30). Prolactin also stimulates lactogenesis in the breast epithelial cells [(26), see section Lactation], but the high progesterone levels during pregnancy inhibit this process, ensuring that lactation does not initiate beforehand [(34), see section The prolactin system. Preparation for lactation]. Progesterone drops after delivery releasing prolactin from inhibition and therefore enabling milk production [(34), see section The prolactin system. Preparation for lactation]. Once established, lactation is sustained by the synchronized action of oxytocin and prolactin, whose secretion is triggered by the baby suckling (30). Prolactin maintains milk production while oxytocin stimulates the milk ejection (30). Aside from their peripheral functions, these hormones are also synthesized and released in the brain, where they act as neuromodulators or neurotransmitters of the maternal brain circuitry (35).

In animals, maternal behavior is triggered by the hormonal events of late pregnancy and parturition (35). Oxytocin and estradiol (E_2_) prime the medial preoptic area (mPOA) region so that maternal behavior emerges. The activation of maternal behavior involves functional plasticity through synaptic strengthening and inhibition, and structural plasticity through modifications of the dendrites, soma size, glial cells, or neurogenesis. These modifications affect critical regions of the maternal circuit (mPOA, hippocampus, olfactory bulb, and PFC) and translate into visible macroscopic structural changes with neuroimaging techniques (36) as well as into behavioral changes.

In humans, the primary approach to investigate how hormones impact the brain is to analyze the women's brain during natural steroid hormonal transitional periods. The common thread of every woman's hormonal transition, either natural (menstrual cycle, puberty, pregnancy and menopause) or assisted (oral contraceptives and hormone replacement therapy) are fluctuations of the E_2_ steroid hormone. Although mainly synthesized by the gonads, E_2_ can also be secreted within many brain regions, including the hippocampus, where it exerts rapid cell-to-cell paracrine effects and regulates memory and learning processes (37). E_2_ fluctuations have been associated with altered neuroplasticity in female rodents across their lifespan (38). In humans, transitional periods characterized by rapid sex-steroid fluctuations such as puberty, the menstrual cycle, contraceptive use, menopause, and hormone therapy are characterized by visible structural and functional modifications of the brain (39, 40). A recent dense-sampling rsfMRI study across one entire menstrual cycle showed that peaks of estradiol confer increased coherence across the brain and increased within-network integration in the DM network (41). The same dense-sampling protocol was repeated under contraceptive use (42). The authors found that high progesterone levels rendered altered GM volumes within medial temporal areas -including the hippocampus- during the natural menstrual cycle, and progesterone suppression induced by contraceptives altered these cycle-dependent effects. Cross-sectional studies have also found interactions between hormonal contraceptive use and GM structure within the right putamen (43), left amygdala, and left parahippocampal regions (44). Notably, all these regions coincide with those where women display structural changes when becoming mothers for the first time (Table 1), suggesting that sex-steroid hormones might modulate DM and subcortical networks activity and structure across a woman's lifespan. Beyond the effects of estradiol and progesterone, pregnancy outcomes -including the emergence of maternal behavior- are indeed the result of a complex interplay between sex-steroid and non-sex-steroid hormones (such as oxytocin and prolactin). For instance, at the end of a rat's pregnancy, estrogen activates mPOA neurons provided that progesterone levels are already low (45). Once primed, this region becomes responsive to oxytocin (46) and prolactin (47), which are essential for the display of maternal behavior and breastfeeding (26, 47). Also, the bursts of oxytocin and prolactin during peripartum are subject to the release of magnocellular neurons and anterior pituitary, respectively, from a tonic progesterone and opioid-mediated inhibition that operates during pregnancy (48). Similar to these pregnancy outcomes, neuroplasticity events occurring during motherhood transition are likely to be mediated by the interaction of different fluctuating hormones.

Adolescence is the transitional period closest to pregnancy in terms of hormonal *milieu*, since both periods are characterized by steady increases in sex-steroid hormones. Moreover, widespread GM brain changes (12) and increased risk for mental health disorders (49, 50) have been described in both transitions. Given these similarities, we hypothesized that a similar profile of brain morphometric changes was operating in pregnancy and adolescence. To test this hypothesis, we compared the longitudinal MR changes that take place in first-time mothers with those occurring in a sample of female adolescents (12). By doing so, we showed that the morphometric changes occurring during the transition to motherhood are comparable in magnitude, shape and direction to those occurring during the adolescence period. Specifically, we quantified the change over time in a detailed set of metrics that fully characterize the brain's anatomy using MRI prospective data of three groups: 25 female adolescents who had never been pregnant, a group of 25 first-time adult mothers, and a group of 20 adult females who had never been pregnant. Both adolescence and pregnancy groups displayed total brain volume reductions together with decreases in cortical thickness, surface area, local gyrification index, sulcal depth, and sulcal length, as well as increases in sulcal width. These findings suggest that the sex-steroid hormonal fluctuations of pregnancy and adolescence exert similar morphometric effects on the cortical mantle.

### Parenting Experience

Although the above literature leads us to consider pregnancy-induced hormones as the primary mediators of maternal brain changes, there are also reasons to think that experience-dependent changes associated with approaching parenthood—which include, but are not restricted to, the interaction with the baby—might also account for the pattern of neural changes observed in mothers. Brain plasticity can also be induced by environmental signals (51). In many rodent species, once the maternal circuit is primed by the endocrine events associated with pregnancy and parturition, maternal behavior is independent of hormonal control and is further maintained by environmental cues derived from the interaction with the offspring [(52) Chapter 3]. When the hormonal events of gestation wane, olfactive and tactile sensory inputs coming from interacting with and nursing the pups stimulate the maternal circuit and maintain maternal behavior. This effect is not limited to dams that go through pregnancy and parturition. In rats, the mere exposure to foster pups through a “sensitization” process converts a female's approach behavior from “avoidant” to “maternal” (53), and co-housed virgin females with dams further boosts the “sensitization” in the virgins to behave maternally (54). Moreover, when retrieving pups, “sensitized” females activate the same hypothalamic hubs that trigger maternal behavior in dams (54) and display signals of neural plasticity (55).

In the human literature, researchers have tried to disentangle the reproductive-induced and experience-induced effects by studying the associations between the degree of maternal brain changes and variables that account for the amount of time the mother spends with her infant. Several studies, including our investigations, have addressed this approach by including in their analyses the time between the birth and the neuroimaging assessment (2, 20). In Hoekzema et al. (2), we did not find associations between the longitudinal structural changes in mothers and the baby age. In contrast, the cross-sectional design of Kim et al. (20) found a positive correlation between the mothers' cortical thickness and the baby age during the first 6 months postpartum. Although it works as an indirect estimate, the baby's age does not accurately indicate how much a mother has interacted with her infant after birth. The amount of mother-infant interaction might be better captured with devices that track the real-time physical proximity between mothers and infants, which are currently under development (56). Complementing such quantitative measures with qualitative assessments of mother-infant interactions such as the “still face” and “strange situation” behavioral tasks would further characterize the quality of that maternal investment.

One other approach to disentangle the reproductive-induced from the experience-induced influence over the parental brain is to analyze the brain adaptations in individuals that experience the parental transition without the reproductive experience. Following that research line, we conducted a longitudinal study in first-time fathers and a group of childless men as a control group (57). We found that fatherhood entails “preconception-to-postpartum” cortical volume and thickness reductions that were positively associated with the father's neural response to pictures of his baby. These reductions were less pronounced and affected fewer parts of the brain compared with those observed in first-time mothers (57). Neural changes in fathers have also been reported during the postpartum period (58) and were different in direction and affected regions compared to those reported in mothers in a similar study by the same group (6). One plausible interpretation of these findings is that the maternal brain changes are mediated by the cumulative effects of the drastic gestational factors and the continued interaction with the baby during postpartum. In contrast, in fathers, the baby's postpartum interaction might be the most relevant operator of the observed neural changes. Another plausible hypothesis is that the parental brain becomes sensitive to postpartum experience factors only in the absence of gestation, similar to what has been described in allomaternal rodent models [(52) Chapter 7]. Finally, researchers are beginning to shift the focus to the expectant parental brain (59). Besides the gestational factors and the parenting experience, the in-pregnancy simulation of certain aspects of parenting is also a candidate factor to trigger the parental brain circuits' remodeling. Indeed, neural responses to infant's stimuli are known to start changing before birth both in mothers (60) and fathers (60, 61), and to predict parenting attunement in the postpartum (62).

### Other Mediating Factors

Beyond parent-infant interaction, there are additional experiences often associated with parenting that might as well contribute to the observed maternal brain changes. 44.5% and 55% of mothers have poor sleep quality during pregnancy and postpartum, respectively (63). A recent study in men and women in their twenties revealed that one night of sleep deprivation was sufficient to induce GM density increases and GM cortical thickness decreases (64). We thus cannot exclude with certainty a partial contribution of perinatal sleep deprivation to the observed GM changes. Stress, either arising from a challenging perinatal environment or from complications during gestation or childbirth, can also influence how women adjust to motherhood (65). Indeed, functional MRI studies find associations between high parenting stress and mothers' brain responses to their infants (66). Future studies should test whether stress disrupts the neuroadaptations to parenting.

Taken together, current evidence suggests that both pregnancy-induced and experience-induced factors can trigger structural changes in the mother's brain. Future studies collecting more specific reproductive-induced (such as hormonal levels) as well as experience-induced factors (such as maternal care, or stress and sleep quality) and comparing the maternal brain adaptations between gestational and non-gestational mothers will help better discern the impact of gestational and environmental factors on the maternal brain.

## What are the Neural Mechanisms?

Our current understanding of the potential neural mechanisms that evoke the macroscopic changes observed during motherhood primarily relies on non-human animal experiments and a few human naturalistic experiments.

In non-human animals, there is extensive evidence that maternal behavior activation involves neural plasticity within several maternal brain regions such as the hippocampus, prefrontal cortex (PFC), basolateral amygdala, nucleus accumbens, and hypothalamus [(52) Chapter 5]. Neural modifications include changes in the number and morphology of neurons and glial cells (astrocytes, oligodendrocytes, and microglia) and synaptic plasticity events that vary depending on the studied species. This warns us that, beyond the similar maternal brain circuits found across mammals, the neural mechanisms behind the maternal brain might not be entirely translatable among species. In humans, inferring neural mechanisms from structural MR imaging is challenging. Although the *ex vivo* human brain has been recently scanned with an ultra-high resolution of 100 μm isotropic (67), the resolution of conventional *in vivo* neuroimaging sequences is still 1 mm isotropic. That resolution can capture major neuroanatomic trends but is insufficient to capture cellular-level processes. These findings imply two things: 1) same neuroanatomic trends could mask different neural processes, and 2) neural processes behind the visible changes are more likely to involve processes that dramatically change the number of brain cells (such as apoptosis or altered proliferation) or the WM myelination, rather than morphological changes in those cells (which are likely to also be happening but would not translate in macroscopic changes). Recalling the human maternal brain literature, two major trends have consistently been found in mothers: pre-to-post pregnancy GM reductions and GM increases during postpartum (Figure 1A). Below we discuss several plausible neural mechanisms through which such GM changes could arise. Reduced proliferation of microglial cells, synaptic pruning, and a myelination process are among the discussed mechanisms. These neural processes are not necessarily mutually exclusive but instead can be operating simultaneously.

During pregnancy, sex-steroid hormones coordinate changes in the mother's immunological system to adapt it to the specific needs of every gestational stage. The three main immune stages are a first pro-inflammatory burst during implantation, followed by an anti-inflammatory state to tolerate the fetus and a final pro-inflammatory peak at parturition (28). The pregnancy-related neuroimmune adaptations extend to the central nervous system, populated by immunocompetent cells known as microglia (68). When activated by pro-inflammatory cues, these cells act as macrophages and remove neuronal debris. A study by Haim et al. (69) showed that, compared to virgin females, dam rats displayed reduced microglial proliferation in maternal brain regions such as the basolateral amygdala, the medial prefrontal cortex, the nucleus accumbens, and the hippocampus prior to parturition and at early postpartum. Notably, the location of such changes is highly resemblant to where GM reductions have been found in mothers (2, 13), suggesting that neuroimmune-mediated microglial reductions might also operate in the human maternal brain. Given their immunosuppressive activity during gestation (70–72), perinatal rising estrogens, progesterone, and glucocorticoids levels are candidates to suppress the proliferation of microglia. In such a case, the pregnancy-induced microglial reduction could buffer the pro-inflammatory activity triggered by the sex-steroid rapid fall after delivery. A reduced microglial coverage would lead to concurrent increases in brain water diffusivity, which has been reported in rats scanned before mating and during pregnancy (73). Diffusion and spectroscopy MRI studies are required to corroborate this microglial phenomenon in human mothers.

Besides their role as immune macrophages, microglia also sculpt neural circuits during brain maturational periods. During adolescence, microglia mediate synaptic pruning by promoting synaptogenesis and engulfing faulty synapses (74–76). Synaptic pruning selectively eliminates redundant synapses during this transition, which is thought to enhance the brain circuitry efficiency in adolescents (77, 78). It has been suggested that the reductions in neuropil and glial elements that surround the pruned synapses, rather than the pruned synapses themselves, are more likely to contribute to the cortical volume and thickness reduction seen in adolescence (79). As previously mentioned, we compared the cortical and sulcal morphometric changes that occur during pregnancy with those occurring during female adolescence and observed the same pattern of morphometric changes in both female samples, that is, a loss of surface area and cortical thickness, and a sulcal widening, causing an overall flattening of the cerebral cortex (12). Based on the similar profile of brain changes and sex-steroid burst between adolescence and pregnancy, we hypothesized that similar neural mechanisms might be behind both life periods.

Rat studies have revealed another pregnancy-induced neuroplasticity event that involves reductions. Physiological events that stimulate oxytocin pulsatile firing (i.e., parturition, lactation, or dehydration) reduce the astrocytic coverage of oxytocin secreting magnocellular neurons in the hypothalamic supraoptic nucleus of rats (80, 81). This increased juxtaposition of nearby neurons facilitates the formation of new synapses, stimulating the contractile (82) and anti-diuretic (83) properties of the oxytocin during birth and lactation. In humans, the small size of the hypothalamus and the lack of clear contrast with its surrounding tissue makes the detection of changes within this region very challenging. Combining more precise automatic segmentation tools of the hypothalamus (84) with higher resolution T1 and T2-weighted MR acquisitions is promising for investigating pregnancy-induced hypothalamic changes.

A third mechanism that might be operating in the observed GM reductions is WM myelination. Pregnant women with demyelinating illnesses such as Multiple Sclerosis (MS) have significantly fewer relapses during the last gestation trimester (85) -when sex-steroid and prolactin hormonal levels are highest-, suggesting that hormonal factors at the end of pregnancy ameliorate the demyelination of this disease. Rodent studies are revealing the mechanism through which such MS improvement might occur. After inflecting WM lesions to pregnant, virgin, and postpartum female rats, Kalakh and Mouihate (86) found less demyelination in pregnant rats compared to the other two groups, an effect that diminished after blocking the dam's GABA receptors or when allopregnanolone (a progesterone metabolite) was antagonized. This study evidenced that the progesterone-mediated GABAergic inhibition that down-regulates the stress axis during pregnancy promotes myelin repair by enhancing the proliferation of oligodendrocytes. The authors also suggested that microglia cells contributed to such re-myelination process by phagocytizing myelin debris. Pregnancy seems to also induce myelination in healthy, free of WM lesions dams. For instance, Gregg et al. (87) found increased oligodendrocytes proliferation during pregnancy, followed by increased axon myelination during postpartum in dams compared to virgin females. Similarly to Kalakh and Mouihate (86), these authors found a pregnancy-induced increased myelin repair capacity after using an acute demyelinating injury. However, Gregg et al. (87) found that prolactin rises -instead of progesterone rises—triggered the observed oligodendrocyte proliferation. Chan et al. (73) found increased whole-brain diffusivity during rat pregnancies, which is a sign of a more diffusion-friendly environment for the water through brain tissues. Based on Gregg et al. (87) and Kalakh and Mouihate (86) findings, it is plausible that pregnancy enhances myelin repair processes, in turn increasing WM integrity, myelination and water diffusivity. However, other processes such as increased extracellular fluid or cell shrinkage might also contribute to these enhanced water diffusion properties. All in all, these rodent studies suggest that pregnancy promotes myelin repair and that both prolactin and progesterone might play an essential role in this process.

Increased myelination can also lead to voxels at the WM and GM interface being misclassified as GM, thus inducing an apparent decrease in cortical volume. It is also plausible that increases in WM are concomitant to the observed cortical GM changes. Together with reporting GM volume and thickness changes, Zhang et al. (3) also detected WM increases within the insula, postcentral gyrus, inferior parietal, and superior and middle temporal gyri during late postpartum. Some of the modified regions overlapped with the GM volume reductions found by the same study (insula and postcentral gyri), while other impacted areas were unique (inferior parietal and superior and middle temporal gyri), suggesting that some GM and WM changes in mothers are simultaneous while some others are independent. Conversely, in Hoekzema et al. (2) we did not detect significant pre-to-post pregnancy changes in WM volume compared to the non-pregnant group. Other than anatomical T1-weighted estimations of WM volume, diffusion MRI provides more precise estimates of WM integrity, fiber orientation, myelin density, and axon diameters (88), which can improve the comprehension of the WM modifications of the maternal brain. Despite being a promising tool, no study has applied this technique to study the human maternal brain during gestation or postpartum so far. The only diffusion-based neuroimaging study in the maternal brain literature is a recent large-scale cross-sectional study with middle-aged mothers (54–81 years) of the UK Biobank neuroimaging database (89). This study revealed that the more parity the less estimated brain age based on their WM characteristics, which suggests a protective effect of parity on WM later in life. However, the cross-sectional nature of the samples does not allow us to discern between the effect of reproductive experience and that of other individual variables.

A last possibility would be that the GM reductions in mothers reflect neurodegenerative processes. E_2_ drop after birth provokes a microglial pro-inflammatory state that can lead to neural injury. It is thus possible that a disbalance of the neuroimmune environment during the motherhood transition could lead to neural injury and predispose for mental health disorders. Indeed, inflammatory levels were ranked as the second-best, after depression history, predictor for postpartum depression levels (90). However, we believe it is unlikely that the changes in GM that occur during a healthy motherhood transition reflect a neurodegenerative process. Pregnant women often report memory deficits while pregnant (91). However, the few cognitive assessments during postpartum have mixed findings: some authors report poorer cognitive performance in postpartum mothers compared to non-mothers (92–94), while other studies have not found any cognitive decline in mothers (95) nor cognitive differences between mothers and non-mothers (95). The reported cognitive impairments could be biased by the physical (i.e., sleep levels) or psychological stressors that come with the motherhood transition, rather than reflecting a neural-mediated cognitive incapacity. In support of this, scholars have recently proposed the “cognitive costs of reproduction model,” which hypothesizes that perinatal cognitive declines result from the mother's temporary reallocation of metabolic and attentional resources to boost the fetus' development and the mother-infant relationship (96). Neuroimaging studies that have compared the mother's brain with the brain of nulliparous women have not found cognitive differences among these two groups (2, 5), and rather suggest that the motherhood-related brain changes are associated with behavioral outcomes that improve the mother's ability to deal with the challenges ahead. Specifically, the degree of structural brain adaptations in mothers predict higher levels of mother's positive perception (6) and attachment (2) to the baby, and stronger functional activation to their babies' signals (13). Also, brain reductions in DM regions observed before and after pregnancy (2) have been argued to reflect an in-pregnancy down-regulation of higher-order cognitive functions in favor of low-order brain functions that provide mothers greater resilience to stress and pain during childbirth (59). Altogether, current results suggest that maternal brain changes reflect a neural adjustment or specialization rather than a neurodegenerative process. Neuroimaging studies with larger and more heterogeneous samples and cognitive measures that directly compare the brain changes in mothers and the brain changes in individuals with neurodegenerative disorders will further clarify these preliminary indications.

In contrast to the GM reductions observed from before to after pregnancy, the postpartum period is characterized by increases in GM volume (4–6, 9, 10). Such postpartum GM increases affect frontal cortical areas and extend to subcortical regions such as the hypothalamus, amygdala, hippocampus, and nucleus accumbens, which together form the subcortical maternal core circuit. The hypothalamus, amygdala and hippocampus, among other subcortical regions, have also been recently detected to increase in volume in mice mothers (36). At a neuronal scale, late gestation and parturient rats display increased cell body size (97) and higher dendritic complexity of hypothalamic mPOA neurons (97), and higher dendritic spine concentration within the anterodorsal medial amygdala (98) and within CA1 and CA3 hippocampal regions (55). At a cellular scale, neurogenesis changes have been consistently found in the hippocampus and olfactory bulb of dams. However, there is no consensual pattern of changes in neurogenesis among animal studies -the results are time-dependent and vary among species- [(99), Important considerations], making the interpretation and translation to the human mother brain very challenging. This comes as no surprise given the differences among species in terms of degree of maturity and mobility (i.e., precociality) of the newborns, and mating and parenting strategies, all of which can result in unique maternal brain adaptations. The widespread GM increases observed in human mothers during postpartum involve multiple cortical and subcortical regions, and therefore are likely to reflect more complex processes. Some authors believe that these postpartum changes might reflect a partial recovery from the reductions during pregnancy (5, 8). Following the above-exposed microglial hypothesis, it might be the case that the observed GM increase reflects a microglial proliferation after the postpartum hormonal environment returns to its “normal” non-inflammatory state. This hypothesis needs to be further tested with neuroimaging modalities that can estimate cellular types such as H_1_ spectroscopy and diffusion MRI.

The human maternal brain literature has mainly relied on T1-weighted images, a modality with limited ability to capture tissue contrasts other than the GM and WM ones. Multimodal MR imaging (that is, combining information from different MR contrasts or modalities) is a promising approach to overcome the limitations of each MR modality and provide a comprehensive non-invasive histological picture of the brain. Complementing T1-weighted MR sessions with other MR contrasts or modalities will inform on different tissue macrostructure aspects such as hippocampal and hypothalamic subfields (through high-resolution T2-weighted sequence), and microstructure aspects such as the types of cells (neuronal or glial) and processes (apoptosis, neurogenesis, differentiation) involved (through ^1^H multivoxel spectroscopy).

In conclusion, motherhood-induced anatomical brain changes are likely to involve simultaneous neural processes affecting both GM and WM tissues, and microglia seems to have a crucial role regulating these processes. Also, distinct neural mechanisms seem to be operating under pregnancy and postpartum periods, resulting in opposite neuroanatomic directional trends. MR advances that increase the anatomical resolution of the images and better determine the microstructural tissue properties are required to elucidate the neural mechanisms behind the macroscopic brain changes observed in mothers.

## Which is the Temporal Course of the Changes?

Another critical research line in the parental brain focuses on determining the temporal course of the motherhood-induced brain changes. Existing longitudinal studies have not yet resolved whether this plasticity emerges during pregnancy, parturition, or postpartum. Also, there are mixed results on whether the mother's brain returns to baseline levels after the first 2 years postpartum or remains altered beyond this critical period of maternal investment. Some studies are even considering that the motherhood-induced changes might be permanent.

There is no solid evidence on when exactly human maternal brain changes start. Rodent studies give us some preliminary insights. Haim et al. (69) analyzed the brain's immune cells of dam rats at the beginning, middle, and end of gestation and found reduced microglial density from late pregnancy extending into the postpartum period. Besides, Barrière et al. (36) scanned longitudinally pregnant mice and found GM increases in subcortical regions key for maternal behavior, some of which started during late gestation (mPOA, bed nucleus of stria terminalis, and paraventricular nucleus), and some of which started after birth (hippocampus and amygdala). Therefore, maternal neural plasticity in rodents is evident already in pregnancy. In humans, the only study that has scanned mothers before, during, and after pregnancy used a small sample size (nine participants, of whose only two underwent an MR before conception), and did not include a control group of nulliparous women (7). They found steady reductions in brain size and increases in ventricular size starting around the second trimester of pregnancy and peaking at parturition. To further validate these findings, researchers need to conduct similar studies with higher sample sizes and to include nulliparous women to control for other potential variables and minimize the noise induced by image acquisition, image processing and statistical analyses. Identifying the onset of the neural remodeling in mothers is necessary to determine when a mother's brain is more vulnerable to environmental stressors (a plastic brain is a vulnerable brain) and would hint at the mediating factors behind the changes.

The other question regarding the temporal course of the changes is whether they are confined to the period of maximal maternal investment or instead persist beyond that period. During the first 2 years of life, infants are highly dependent and require substantial parental investment. This period is equivalent to the “pre-weaning” period of rodents. Several studies using diverse timeframes have detected GM changes in mothers from birth until 2 years postpartum, but most of the studies have not explored the maternal brain beyond that “weaning” period. Literature in rodents indicates that reproductive experience confers both long-term behavioral and neural changes that are evident beyond weaning, and short-term neural changes that restore at late postpartum. On the one hand, post-weaned dam rats are less anxious and fearful than virgin rats and have better foraging strategies (100, 101), and spatial learning and memory skills (100–102). At the neural level, aged parous rats present modifications in hippocampal plasticity (103, 104) and estrogen sensitivity (105) and less markers of brain aging (102). On the other hand, microglia density changes detected in rats during late gestation and early postpartum return to baseline levels after weaning (69). In humans, similar processes are likely to happen: some neural changes might be lasting while others not [reviewed in Duarte-Guterman et al. (106)]. With a notable difference: unlike the rest of the non-human altricial species, human maternal behavior extends beyond the “weaning” period and is often everlasting. Therefore, more long-term neural modifications are expected in human mothers compared to any other animal species.

Trying to fill this gap of knowledge, in a recent study we followed the primiparous mothers of the study of Hoekzema et al. (2) and scanned seven of them again at 6 years postpartum (11). We compared the brains of the primiparous and nulliparous women before conception, during early postpartum and at 6 years postpartum, restricting the analysis to those regions that underwent GM volume reductions between the pre- and post-pregnancy sessions (2). We found that the brain of a mother is still different from that of a nulliparous woman even at 6 years after delivery. Specifically, most of the GM volume reductions were found to persist at 6 years postpartum, being able to classify women as having been pregnant or not with a 91.67% of total accuracy. These preliminary findings suggest that the pregnancy-induced brain changes are long-lasting and open the possibility that they are indeed permanent. There are two plausible explanations for these enduring effects of motherhood on the brain. On the one hand, these enduring brain changes could be due to the organizational effects of hormones during pregnancy and peripartum periods (meaning that the effects last beyond the period of hormonal exposure). On the other hand, other plausible mediators of the motherhood's enduring GM effects might be the long-term alertness and sleep disruption that the parents often experience.

Other brain regions have been found to change during pregnancy but fully or partially reverse to baseline levels after the immediate postpartum. The gradual increase of the pituitary gland observed during pregnancy returns to normal size coinciding with the end of breastfeeding (16, 17), a finding that was further replicated by Hoekzema et al. [(2); Supplementary Figure 3]. Hoekzema et al. (2) also found that, while all regions that displayed volume changes remained reduced up to 2 years postpartum, the GM reductions within the hippocampus partially recovered at the 2 year postpartum visit.

Recent investigations have approached the long-term effects of parenthood by analyzing how elderly subjects who became parents decades ago differ neuroanatomically from those who did not have children. In elder women, a higher number of previous children has been found to be associated with less apparent brain aging in WM (89), cortical (89, 107) and subcortical regions (108), larger global GM cortical volume (89) and thickness (109) and distinct patterns of resting state functional connectivity (110). In Ning et al. (111), middle-aged females and males with a higher number of children displayed better visual memory, faster response time, and lower predicted brain age, suggesting that lifestyles associated with parenting, rather than the physiology of pregnancy and lactation themselves, might be beneficial for brain aging processes. Altogether, these studies suggest that motherhood exerts neural changes that persist into older age.

Given these findings, we believe it is necessary that human neuroimaging databases and studies that track brain trajectories across lifespan start including parity-related variables as part of the participants' relevant demographic information.

## Do These Structural Changes Have an Implication in Postpartum Mental Health?

The physiological and endocrine signals around the peripartum ensure labor and help establish lactation, while putting the mother at risk for developing mental health disorders. Placental separation at birth causes rapid progesterone and estradiol withdrawal, whose levels remain low during the early postpartum (112). This rapid drop triggers neurochemical deficiency in the serotonin and dopamine systems and affects the GABAergic inhibitory system, a neurochemical pattern that resembles a depression-like scenario [discussed in Sacher et al. (113)].

Postpartum mood changes resulting from such hormonal changes and increased stress and loss of sleep are expected in mothers after birth, but one in every five postpartum mothers experience a much more severe disorder: postpartum depression (PPD) (50). In the Diagnostic and Statistical Manual of Mental Disorders (DSM 5th edition) (114), PPD is still vaguely defined as a “major depression event with peripartum onset.” However, beyond the unique timing of PPD, this disorder involves a different symptomatology, often characterized by avoidance, intrusive behaviors or even psychosis toward the baby. If left unrecognized or untreated, the disorder can have serious consequences for both the mother and the infant's well-being (115). Unfortunately, in spite of the importance of early PPD identification and treatment, PPD is still often unrecognized in mothers.

Neuroscience researchers are bringing efforts to identify the unique neurobiological and pharmacokinetic profiles of PPD, and numerous authors have reviewed the neuroimaging literature on the topic (113, 116–118). In humans, functional MR Imaging is the current preferred choice of study of PPD. A wide range of task-based functional studies have compared the neural activations of mothers with PPD diagnosis (or depression predisposition) and non-depressed mothers upon infant stimuli. Given its vast implication in depression symptomatology, most studies chose the amygdala as the seed region of interest (119) followed by the PFC (119, 120) anterior cingulate cortex (120) and ventral striatum (119). In contrast, two studies explored activations at the whole-brain level (121, 122). Studies usually compare the pattern of brain activation while exposing mothers to cues of their baby vs. of unrelated babies (121–125), although some studies have also used as stimuli unrelated infants crying (120), images of adult faces (119), and emotionally valenced words (126, 127). These studies also vary in the proportion of primiparous and unmedicated mothers, diagnosis criteria, and postpartum time-points assessed. Still, most of the analyses find differential BOLD activation within the amygdala, anterior cingulate cortex, prefrontal cortex, insula and ventral (nucleus accumbens), and dorsal (caudate and putamen) striatum, and disrupted connectivity between the amygdala and the insula (123), nucleus accumbens and anterior cingulate regions (120). Notably, these structures coincide with the changing regions reported in Table 1. Wonch et al. (123) found that mothers' amygdala response and functional connectivity between the amygdala and insula differed between primiparous and experienced mothers, stressing the importance of studying primiparity and multiparity separately. Dudin et al. (124) were the first to report differences in amygdala responsivity between mothers with PPD and women with major depression, using as controls non-depressed mothers and non-depressed women. This study was pioneer in characterizing the unique neural profiles of depression during the perinatal period compared to depression arousals at other times in a woman's life, which has been reviewed by Pawluski et al. (128).

Maternal brain researchers have also applied rsfMRI to capture the basal patterns of disrupted functional connectivity in mothers with PPD. Given that this MRI modality does not require the cognitive engagement of the mothers while scanning, it offers unique insights into the neuroscience behind PPD. Studies investigating PPD through rsfMRI are diverse in analyses but have consistently explored connectivity patterns from the amygdala and cortical DM nodes. Besides, unlike task-based functional MRI studies, all resting-state studies have excluded medicated mothers to remove potential pharmacological interactions with the results. Five studies explored resting-state functional connectivity patterns in depressed and non-depressed mothers at the whole-brain level (129) or using seed analyses from the posterior cingulate cortex (130, 131), the anterior cingulate cortex, amygdala, hippocampus, and dorsolateral prefrontal cortex (dlPFC) (132), and the dorsomedial prefrontal cortex (dmPFC) (133). These studies found information flow direction changes (129), reduced interhemispheric connectivity (131), and altered (increased, decreased or opposing) functional connectivity (132, 133) among the seed regions (132), between the seed regions and DM cortical nodes (133), thalamus, caudate (132) and amygdala (130), and among DM cortical nodes (129–131) and the amygdala (129). Two other studies analyzed changes in the regional or local resting-state homogeneity in PPD and non-PPD mothers through Regional Homogeneity (ReHo) or fractional Amplitude of Low Frequency Fluctuation (fALFF) methods (134, 135), rendering mixed results of increased and decreased DM regional homogeneity in PPD mothers compared to healthy mothers. The depression scores of mothers with PPD correlated with the regional (135), interhemispheric (131), and DM connectivity of the dmPFC (131, 133) or the dlPFC (135), suggesting that functional connectivity in this region is especially relevant in the severity of PPD.

As observed, task-based and resting-state studies in mothers with PPD find functional disruptions in regions that match with the frontal, temporal, and cingulate cortical regions and subcortical regions (amygdala, hippocampus, thalamus and striatum) that are structurally modified in healthy mothers (Table 1), suggesting that PPD might disrupt neuroanatomic trajectories during motherhood transition. Despite this significant overlap, no human study to date has analyzed the brain structure of mothers with PPD. Instead, human studies that track brain structural trajectories across motherhood have focused on delineating the healthy pattern of brain adaptations. The pre-to-post pregnancy sample of Hoekzema et al. (2) (*N* = 25) only included one mom that developed postpartum depression, and results were similar after excluding that woman from the analysis. Longitudinal studies during the postpartum have excluded those mothers with ongoing psychiatric conditions (3, 8) or have relied on mothers with minimal to moderate levels of depression [ <13 Beck's Depression Inventory score in Kim et al. (6); <10 Edinburgh Postnatal Depression Scale score in Lisofsky et al. (5)]. Moreover, in those studies that did include depression questionnaires, mothers and non-mothers did not differ in depression levels, and thus the studies did not assess structural changes in relation to this variable (2, 5). The lack of PPD representation in structural MRI studies has a plausible reason why: the neuroadaptations across healthy motherhood are still unresolved, and to unravel them, studies prioritize minimizing the confounders in their analyses. We believe that a grounded comprehension of the typical trajectories of change in healthy mothers will frame sound hypotheses on which patterns of disruptions are expected in mothers with postpartum depression. Nonetheless, maternal brain neuroimaging studies must start including the depression and anxiety levels of the participants routinely and exploring the neuroanatomic adaptations in relation to such levels. Postpartum mental health needs to be explored in relation to past or present complications during pregnancy. Also, future studies should explore the implications of different birth courses. Parturitions vary in terms of the degree of intervention, medicalization, and the mother's psychological and physiological experience, and some of these events can impact the mother's mental health (136–139) and their neural response and bonding toward the infant (140, 141). Finally, uncovering the risk factors of this disease is paramount to developing prediction and early intervention strategies. Previous history of mental illness and stress, life-long vulnerability to hormonal changes (including premenstrual disorders and response to hormonal treatments or contraceptives), and perinatal poor sleep quality are considered risk factors for developing PPD (113). These variables should be tested as potential predictors for deviant brain morphometric trajectories during the transition to motherhood.

## Conclusions

Our understanding of the human maternal brain has grown considerably during the last 20 years. Before the twentieth century, scholars established the neuroendocrinology of maternal behavior in rodents (52), and there were first indications that pregnancy modified the pituitary gland of human mothers (15–18). Inspired by these initial works, new-age maternal brain researchers have proved groundbreaking evidence that gestation impacts the mother's brain at a structural and functional level. Besides, recent brain age prediction studies suggest that parity also affects the long-term trajectories of brain aging. Here, we have reviewed and discussed the current evidence about the trajectories, mediating factors, and neural mechanisms behind the human maternal brain remodeling and its potential relation to postpartum mental health. As argued alongside the review, the answers to these questions are not entirely resolved, and the function that the brain structural changes observed in mothers might serve is still under debate. Through longitudinal neuroimaging designs with first-time parents, our research group has found preliminary evidence that maternal brain changes are pronounced (2) and long-lasting (2, 11), that involve a flattening of the cortex similar to the one occurring during adolescence (12), that predict mother-to-infant attachment (2), and that can be mediated by pregnancy-induced factors as well as by the parental experience with the infant (12, 57). Future investigations are needed to shed light on this nascent but exciting research area, especially studies with larger and more diverse samples in terms of culture and socioeconomic status, as well as those examining parents at multiple time points of their parental transition.

## Author Contributions

MM-G, SC, and MP-D contributed to the conception and design of the review. MM-G wrote the first drafts of the manuscript. SC designed the figure. All authors contributed to manuscript revision, read, and approved the submitted version.

## Funding

This work was supported by Ministerio de Ciencia e Innovación project RTI2018-093952-B-100 and by Instituto de Salud Carlos III projects CP16/00096 and PI17/00064, and co-funded by European Regional Development Fund (ERDF), A way to make Europe. MM-G was funded by Ministerio de Ciencia e Innovación, Instituto de Salud Carlos III, Predoctorales de Formación en Investigación en Salud (PFIS) (contract FI18/00255) and a predoctoral Fulbright grant. SC was funded by a Miguel Servet Type I research contract (CP16/00096). MM-G and SC were co-funded by European Social Fund Investing in your future. The project leading to these results has received funding from la Caixa Foundation under the project code LCF/PR/HR19/52160001, from the European Research Council (ERC) under the European Union's Horizon 2020 research and innovation programme (grant agreement No 883069), and from the Centro Nacional de Investigaciones Cardiovasculares (CNIC). The CNIC is supported by Instituto de Salud Carlos III (ISCIII), Ministerio de Ciencia e Innovación and the Pro CNIC Foundation, and is a Severo Ochoa Center of Excellence (SEV-2015-050).

## Conflict of Interest

The authors declare that the research was conducted in the absence of any commercial or financial relationships that could be construed as a potential conflict of interest.

## Publisher's Note

All claims expressed in this article are solely those of the authors and do not necessarily represent those of their affiliated organizations, or those of the publisher, the editors and the reviewers. Any product that may be evaluated in this article, or claim that may be made by its manufacturer, is not guaranteed or endorsed by the publisher.
